# Testing Longitudinal Measurement Invariance of the Dutch PSS-10, MBI-ES, and PANAS in a Bimonthly 6-Month Panel

**DOI:** 10.5334/pb.1528

**Published:** 2026-05-25

**Authors:** Justine Van de Velde, Bert Weijters, Katia Levecque

**Affiliations:** 1 ECOOM HR in R&D, Belgium; 2 Department of Work, Organization and Society, Faculty of Psychology and Educational Sciences, Ghent University, Belgium

**Keywords:** longitudinal measurement invariance, stress, emotional exhaustion, burnout, affect, teachers

## Abstract

Monitoring teacher well-being over time is essential in both research and practice to understand changes, evaluate interventions, and support occupational health. This study examines the longitudinal measurement invariance of the Dutch versions of three commonly used scales related to well-being: the Perceived Stress Scale (PSS-10), the Emotional Exhaustion subscale of the Maslach Burnout Inventory (MBI), and the Positive and Negative Affect Schedule (PANAS). Utilizing three bimonthly waves of data collected from a cohort of elementary school teachers in Flanders (n = 237), we evaluated the scales’ configural, metric, scalar, and residual invariance over time. Confirmatory factor analyses indicated that all three scales demonstrate adequate longitudinal measurement invariance, supporting their stability and reliability for repeated measurement. Notably, this is among the first studies to test longitudinal measurement invariance of these widely used scales over a 6-month period in an applied occupational setting. These findings strengthen confidence in their use for monitoring well-being over time.

Monitoring well-being over time in occupational settings has become increasingly important for both research and practice. Understanding how employees’ psychological states evolve can provide crucial insights into individual functioning, organizational dynamics, and the effectiveness of interventions aimed at promoting mental health at work (Sonnentag & Frese, 2012). Well-being is not a static condition. Rather, it fluctuates over time due to changes in job demands, resources, and personal circumstances (Demerouti et al., 2014). But to capture such fluctuations meaningfully, measurement itself must be sound; otherwise apparent changes may reflect nothing more than unintended measurement effects (Baumgartner & Steenkamp, 2006; Widaman et al. 2010).

In educational contexts, the validity of repeated assessments is a central concern, particularly when instruments are used to monitor teacher well-being, inform school-level interventions, or evaluate professional-development programs. As such, establishing longitudinal measurement invariance is essential to ensure that changes reflect true score variation rather than shifts in measurement functioning.

Among the many dimensions of well-being, perceived stress, emotional exhaustion, and affect are widely recognized as core constructs. Perceived stress reflects the extent to which individuals feel that their lives are unpredictable, uncontrollable, and overwhelming, and it has been linked to numerous health and performance outcomes (Cohen et al., 1983; Cohen et al., 1993). Emotional exhaustion represents the feeling of being emotionally overextended and depleted of emotional resources, and it is often regarded as the central component of burnout (Maslach et al., 2001; Schaufeli et al., 2009). Positive and negative affect reflect the extent to which individuals experience pleasurable or unpleasurable mood states, respectively, and these two dimensions have been shown to be relatively independent and predictive of a range of occupational and health-related outcomes (Watson et al., 1988; Pressman & Cohen, 2005). Despite their widespread use in educational and occupational research, these instruments are rarely examined for invariance across repeated assessments in teacher samples, leaving open the question of whether observed changes represent meaningful psychological change or artefactual measurement drift. Teachers are an especially relevant population in this regard, as their well-being is increasingly monitored in schools and educational research. Ensuring the longitudinal equivalence of the instruments used in these contexts is therefore crucial for accurate interpretation.

To assess these constructs, the Perceived Stress Scale (PSS-10), the Emotional Exhaustion subscale of the Maslach Burnout Inventory (MBI-ES), and the Positive and Negative Affect Schedule (PANAS) are among the most widely used and validated self-report instruments. These measures have demonstrated good psychometric properties across a variety of populations and cultural contexts (Lee, 2012; Maslach et al., 2016; Merz et al., 2013). However, despite their widespread use, relatively little research has examined whether these instruments maintain their measurement properties when applied longitudinally.

The present study addresses this gap by systematically evaluating the longitudinal measurement invariance (LMI) of the Dutch versions of these three scales across three bimonthly waves in an occupational sample. By establishing whether these commonly used measures retain their validity over time, the study contributes to more rigorous monitoring of psychological well-being in applied settings.

## Longitudinal measurement invariance

*Measurement invariance*, or *measurement equivalence*, is a statistical property of a measurement instrument that implies that the instrument measures the same construct in the same way across different contexts (e.g., groups or time points) (Davidov et al., 2014; Steenkamp & Baumgartner 1998). Although measurement invariance can be evaluated across groups or over time, most empirical applications have focused on cross-sectional comparisons, particularly cultural or demographic groups (Alatlı 2020). Longitudinal applications have received comparatively less attention (Alatlı 2020; Van de Schoot et al., 2015), and are often bypassed altogether in favor of directly comparing observed or latent means.

The focus of the current study is on LMI, also referred to as measurement invariance over time or temporal measurement invariance, which concerns the degree to which a measurement instrument assesses the same construct in the same way across repeated measurement occasions (Widaman, Ferrer, & Conger, 2010). Establishing this property is essential for interpreting observed score changes as true changes in the underlying construct rather than as artifacts of shifts in measurement properties (Van de Schoot et al., 2015).

When LMI is not met, apparent changes may instead reflect alterations in the meaning or functioning of items over time. For example, in a clinical setting Fried et al. (2016) found that several widely used depression scales failed to meet LMI, with changes in factor structure and item parameters across assessments. As a result, decreases in total scores could not be interpreted unambiguously as improvements in depression. To avoid such problems, the recommended approach is to test LMI using longitudinal confirmatory factor analysis, which allows for a systematic evaluation of the stability of measurement parameters over time.

**Figure 1:**
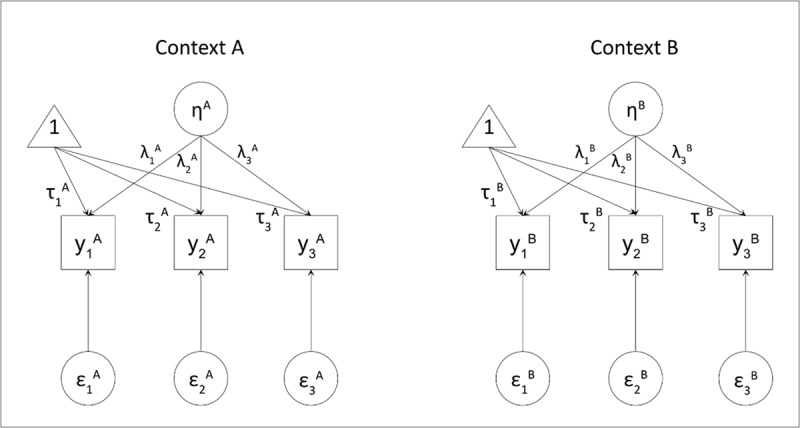
Confirmatory Factor Analysis of Construct *η* at time points A and B, with Indicators *y_i_*, Factor Loadings *λ_i_*, Item Intercepts *τ_i_*, and Item Error Variances Var(*ɛ_i_*). *Note*. A and B refer to two time points. To avoid clutter, the covariances between the latent variables and between the time-specific observations of the items are not displayed.

LMI is evaluated in a hierarchical sequence, with each level building on the previous one (Widaman et al., 2010; Van de Schoot et al., 2015; Steenkamp & Baumgartner, 1998). Figure 1 displays a factor model for one construct measured at two points in time to help illustrate these levels of LMI. *Configural invariance* is the least restrictive level and requires that the same factor structure holds at each measurement occasion. This means the same number of factors and the same pattern of relationships between factors and indicators should be observed. Configural invariance establishes that the construct is conceptualized similarly over time, which is a prerequisite for any longitudinal comparison. If this holds, *metric invariance* (also called weak invariance) tests whether factor loadings (λ) are equal across time points. Equal loadings ensure that the scale of the latent construct is the same over time, allowing for valid comparisons of associations between constructs (e.g., covariances, regression coefficients) across time. The next level, *scalar invariance* (strong invariance), tests whether item intercepts (τ) are equal over time. This is required to compare latent means meaningfully; without scalar invariance, observed mean differences may reflect shifts in item calibration rather than true change in the construct. Finally, *residual invariance* (strict invariance) tests whether item residual variances (Var(ɛ)) are equivalent over time. While less critical for many substantive analyses, residual invariance indicates that the amount of measurement error is stable, which supports more precise comparisons and can improve the accuracy of structural parameter estimates.

In practice, the strict requirements of full measurement invariance are often difficult to achieve, especially in longitudinal contexts where repeated exposure to items, developmental processes, or contextual changes can influence how respondents interpret or respond to particular indicators over time. In such cases, enforcing full metric or scalar invariance may result in poor model fit, leading researchers to reject LMI altogether. However, a lack of full invariance does not necessarily preclude meaningful comparisons. This is where *partial measurement invariance* becomes essential.

Partial LMI refers to a model in which a subset, but not all, of the measurement parameters (e.g., loadings, intercepts) are constrained to equality over time, while others are allowed to vary (Byrne, Shavelson, & Muthén, 1989). The approach assumes that sufficient invariance exists to justify substantive comparisons, even if some items behave differently across time points. For instance, scalar invariance may still be established if most item intercepts are invariant, and the non-invariant parameters are either theoretically justified or empirically identified and allowed to vary freely (Steenkamp & Baumgartner, 1998; Marsh et al., 2010).

To implement partial invariance, researchers typically follow a stepwise testing approach. When full invariance is rejected, modification indices or parameter change statistics can guide the identification of non-invariant items. These constraints can then be selectively relaxed to improve model fit. Importantly, decisions to relax constraints should be informed not only by statistical indices but also by substantive theory and an understanding of the construct being measured (van de Schoot et al., 2015). Arbitrary freeing of parameters risks capitalizing on sample-specific noise and undermines the interpretability of longitudinal comparisons.

Simulation studies suggest that valid comparisons of latent means and structural relationships are still possible under partial scalar or metric invariance, provided that at least two indicators per construct maintain invariance (Steinmetz, 2013). Nevertheless, it is important to report the extent and nature of non-invariance, to qualify inferences and assess the robustness of conclusions.

Partial invariance thus provides a pragmatic middle ground between the ideal of full invariance and the practical realities of measurement in longitudinal research. It enables researchers to retain most of the benefits of measurement invariance testing (more valid inferences about change) while acknowledging and accommodating imperfections in measurement over time.

## LMI of three worldwide used well-being scales: PSS, MBI, and PANAS

Well-being is a complex and multidimensional construct for which numerous measurement scales have been developed. Because well-being and its dimensions may manifest differently across groups, contexts, or points in time, it is essential to use scales that demonstrate measurement invariance. LMI is particularly important in trend research and panel surveys, as respondents’ conceptualization of well-being may change over time.

In this study, we focus on three well-being scales that are widely used across the world: the Perceived Stress Scale (PSS), the Maslach Burnout Inventory (MBI), and the Positive and Negative Affect Schedule (PANAS). For these scales, most existing measurement invariance studies have examined invariance across gender (for the PSS-10, see Chen et al., 2024; for the MBI, see Aboagye et al., 2018; for the PANAS, see Ortuño-Sierra et al., 2015). However, relatively little attention has been given to the *longitudinal* equivalence of these scales.

Table 1 presents an overview of the state of the art.

**Table 1:** Studies Investigating the LMI of the PSS-10, MBI, and PANAS.


FIRST AUTHOR, YEAR OF PUBLICATION, TITLE	SCALE VERSION	POPULATION AND LOCATION	DESIGN	*N*	LMI LEVELS TESTED	LMI LEVELS SUPPORTED

Jiang et al. (2023): Validation of the Chinese version of the Perceived Stress Scale-10 integrating exploratory graph analysis and confirmatory factor analysis	PSS-10, Chinese	University students, China	2 waves, 1-week interval	492	Configural Metric Scalar Residual	Configural Metric Scalar Residual

Reis et al. (2019): The German Version of the Perceived Stress Scale (PSS-10): Evaluation of Dimensionality, Validity, and Measurement Invariance With Exploratory and Confirmatory Bifactor Modeling	PSS-10, German	Adult participants of six RCTs testing the same stress management training, Germany	2 waves, 8-week interval	1862	Configural Metric Scalar	Configural Metric Scalar

Isik Akin et al. (2024): Parent-Child Relationship, Well-Being and Home-Leaving during the Transition from High School to University	PSS-10, Turkish	University students, Turkey	2 waves, 18-month interval	240	Configural Metric Scalar	Configural Metric Scalar

Shields et al. (2016): Stress-related changes in personality: A longitudinal study of perceived stress and trait pessimism	PSS-10, English	Young, middle, and older adults from college campuses and surrounding community, USA	5 waves, 1-week intervals	332	Configural Metric Scalar	Configural Metric Scalar

Barbosa-Leiker et al. (2013): Measurement Invariance of the Perceived Stress Scale and Latent Mean Differences across Gender and Time	PSS-10, English	Participants of the Spokane Heart Study: asymptomatic, community-dwelling adults, USA	2 waves, 2-year interval	871	Configural Metric Scalar	Configural Metric Scalar

Turhan et al. (2021): Psychometric Properties of the German Short Version of the Maslach Burnout Inventory - Student Survey	MBI short student version, German	University students, Germany	3 waves, 6-week intervals	1435	Configural Metric Scalar Residual	Configural Metric Scalar Residual

Giusti et al. (2022): The North Italian Longitudinal Study Assessing the Mental Health Effects of SARS-CoV-2 Pandemic Health Care Workers—Part II: Structural Validity of Scales Assessing Mental Health.	MBI, Italian	University hospital staff, Italy	2 waves, 16-month interval	369	Configural Metric Scalar	Configural Metric

Kim et al. (2009): Factor Structure and Longitudinal Invariance of the Maslach Burnout Inventory	MBI, English	Social workers, USA	2 waves, 1-year interval	282	Configural Metric	Configural Metric (partial)

Mäkikangas et al. (2011): Longitudinal factorial invariance of the Maslach Burnout Inventory-General Survey among employees with job-related psychological health problems	MBI, Finnish	Employees who participated in two different rehabilitation intervention programs, Finland	2 waves, 4-month interval	155	Configural Metric Residual	Configural Metric Residual

Schnitker et al. (2020): Bidirectional associations across time between entitativity, positive affect, generosity, and religiousness in adolescents training with a religiously affiliated charity marathon team	PANAS adapted for children, English	Adolescents and emerging adults from running teams, USA	3 waves, 15-week interval between T1 and T2, 3-week interval between T2 and T3	396	Configural Metric Scalar	Configural Metric Scalar

Jønsson et al. (2022): The savvy and cheerful employee innovation champions: The roles of political skill and trait-positive affect in employees’ championing and salary levels	Positive affect items of the PANAS short form, Danish	Employees from a branch of a global IT consultancy company, Denmark	3 waves, 4-month intervals	152	Configural Metric Scalar	Configural Metric Scalar

Belkin et al. (2022): Beliefs in government benevolence can promote individuals’ compliance with government-issued guidelines: The role of positive affect and general construal level	Positive affect items of the PANAS short form, English	Adult Mechanical Turk participants, USA	2 waves, 2-week interval	195	Configural Metric Scalar	Configural Metric Scalar

Xiang et al. (2023): Development of self-concept clarity from ages 11 to 24: Latent growth models of Chinese adolescents	Positive Affect subscale of the PANAS, Chinese	Adolescents, China	3 waves, 6-month intervals	2001	Configural Metric Scalar	Configural Metric


### Perceived Stress Scale

Cohen et al. (1983) originally developed the Perceived Stress Scale (PSS) to measure the degree to which individuals perceive their lives as stressful, unpredictable, uncontrollable, and overwhelming. The earliest version consisted of 14 items (PSS-14), but shorter versions with ten items (PSS-10) and four items (PSS-4) were later validated (Cohen & Williamson, 1988). Lee (2012) reviewed the literature on the validity of all three versions and concluded that the PSS-10 has the best psychometric properties. The PSS-4 was judged to be the least effective, although it remains useful in situations requiring a very brief measure, such as telephone interviews.

All versions include both positively and negatively worded items. The developers recommend reversing the scores for positively worded items and calculating a single perceived stress score (Cohen et al., 1983; Cohen & Williamson, 1988). However, the one-dimensionality of the scale has been questioned, with empirical evidence suggesting that a two-dimensional structure may better represent the data (Lee & Jeong, 2019; Tikka et al., 2022; Yılmaz Koğar & Koğar, 2024).

The PSS-10 has become one of the most widely used measures of psychological stress and has been translated into many languages, including Chinese, Hindi, Spanish, French, Arabic, German, and Turkish. The Dutch translation used in the present study was developed for the Longitudinal Aging Study Amsterdam (Korten et al., 2017; van Eck et al., 1996) and has been applied in multiple studies in the Netherlands and in Flanders, the Dutch-speaking region of Belgium (Ceulemans et al., 2021; Van Kerkhoven et al., 2022). These studies followed the developers’ recommended scoring procedure, producing total perceived stress scores.

LMI of the PSS-10 has been evaluated for the Chinese (Jiang et al., 2023), German (Reis et al., 2019), Turkish (Isik Akin et al., 2024), and English versions (Barbosa-Leiker et al., 2013; Shields et al., 2016). Table 1 summarizes the main characteristics of these studies. All versions demonstrated full metric and scalar invariance. Notably, the Chinese study was the first to also confirm residual invariance. These studies used either student samples (Isik Akin et al., 2024; Jiang et al., 2023; Shields et al., 2016) or community samples (Barbosa-Leiker et al., 2013; Reis et al., 2019; Shields et al., 2016). Most designs involved two waves of data collection with either short (1–8 weeks) or long (18–24 months) intervals. Shields et al. (2016) used five waves, but with one-week intervals. The present study extends this literature by examining all four levels of LMI in an employee sample, using three waves spaced two months apart.

### Maslach Burnout Inventory

The Maslach Burnout Inventory (MBI) measures three core dimensions of burnout: emotional exhaustion, depersonalization, and reduced personal accomplishment (Maslach & Jackson, 1986). The scale contains three subscales, one for each dimension. The developers recommend that these subscales not be combined into a single burnout score; instead, separate scores should be calculated for each dimension. Emotional exhaustion is often considered the central component. Chronic stress is thought to deplete an individual’s emotional resources, leading to feelings of being emotionally drained and ultimately to burnout (Maslach & Jackson, 1986).

The concept of burnout and the original MBI were developed for human services occupations (Maslach Burnout Inventory–Human Services Survey; MBI-HSS). The instrument was later adapted for non-human services occupations (Maslach Burnout Inventory–General Survey; MBI-GS), for educators (Maslach Burnout Inventory, Educators Survey; MBI-ES), and for students (Maslach Burnout Inventory–Student Survey; MBI-SS; Schaufeli et al., 2002).

The MBI is the most widely used burnout measure worldwide and has been translated into many languages, including Chinese, Spanish, French, Arabic, Finnish, German, and Italian. The Dutch translation of the MBI-ES, produced by Soenens et al. (2012), is used in the present study. In Flanders, multiple studies have employed this version, either by computing subscale scores or by focusing on a single subscale (Aelterman et al., 2019; Hellebaut et al., 2023; Van den Berghe et al., 2013, 2014; Vermote et al., 2023).

LMI of the MBI has been examined for the Finnish (Mäkikangas et al., 2011), German (Turhan et al., 2021), and Italian (Giusti et al., 2022) versions. Table 1 summarizes the main characteristics of these studies. All three reported full metric invariance, though results for scalar and residual invariance were mixed. The German version showed full scalar and residual invariance. The Finnish study did not test scalar invariance but reported residual invariance. The Italian study found no support for scalar invariance and did not test residual invariance. Samples included students (Turhan et al., 2021) and employees (Giusti et al., 2022; Mäkikangas et al., 2011). The Finnish and Italian studies used two waves with intervals of four and sixteen months, respectively; the German study used three assessments six weeks apart. The present study adds to this literature by focusing on a high-risk occupational group, teachers, and examining all four levels of LMI across three waves spaced two months apart.

### Positive and Negative Affect Schedule

Watson et al. (1988) developed the Positive and Negative Affect Schedule (PANAS) to measure two dominant dimensions of affect: positive affect and negative affect. Positive affect reflects energy, concentration, and pleasurable engagement, illustrated by emotions such as enthusiasm, activity, and alertness. Negative affect reflects unpleasurable engagement or subjective distress, exemplified by emotions such as anger, contempt, and guilt.

The PANAS contains two separate subscales, Positive Affect and Negative Affect, which are scored independently. Meta-analytical work confirms that the PANAS is best conceptualized as two distinct but correlated factors (Wedderhoff et al., 2021), with correlated error terms informed by the content categories proposed by Zevon and Tellegen (1982). The instrument has been shown to have strong reliability and validity (Crawford & Henry, 2004).

The PANAS has been translated into numerous languages, including Chinese, Spanish, French, and Danish, making it a widely used measure across cultures. The Dutch version used in the present study has been applied in several Flemish studies (Brenning et al., 2017; Engelen et al., 2006; Laporte et al., 2021, 2022). In these studies, the two subscales were used independently: in some cases only one was administered, while in others both were included but scored separately, following the recommendations of Watson et al. (1988).

Table 1 summarizes the main characteristics of studies on the LMI of the PANAS. Only one has examined LMI for the Positive Affect subscale (Xiang et al., 2023), finding configural and metric invariance but scalar non-invariance in a sample of Chinese adolescents. Adapted short forms of the Positive Affect subscale have also been studied. A Danish study with a four-item version (Jønsson & Kähler, 2022) and an English study with a five-item version (Belkin & Kong, 2022) both found full configural, metric, and scalar invariance in adult samples. A study on the children’s version of the Positive Affect subscale also found full configural, metric, and scalar invariance in American adolescents (Schnitker et al., 2020).

Three of these studies used three waves of data collection with intervals of four to six months (Jønsson & Kähler, 2022; Schnitker et al., 2020; Xiang et al., 2023). The Mechanical Turk study used two waves two weeks apart (Belkin & Kong, 2022). None tested residual invariance, and no study has examined the LMI of the Negative Affect subscale. The present study addresses both gaps by testing all four levels of LMI for both subscales of the original full-length PANAS in an adult employee sample.

## Method

### Participants and procedure

This study uses data from a longitudinal project examining the impact of meditation on the workplace well-being of elementary school teachers in Flanders and Brussels. In this project, teachers practiced meditation with their pupils every school day for six months. Three waves of data collection monitored well-being of the intervention group and a waitlist control group. At each wave, the same set of well-being scales was administered to capture multiple dimensions of well-being over time. All participants (intervention and control) completed three surveys: the first in January 2021 (T1), the second in March 2021 (T2), and the third in June 2021 (T3). More information about the project is available elsewhere (ISRCTN61170784). The study, including informed consent, was approved by the Ethical Committee of Ghent University (2020/164). Given the sensitive nature and the lack of explicit participant consent with regards to sharing, the data cannot be made publicly accessible.

The sample consisted of 237 participants, of whom 216 were female (91.9%) and 21 were male (8.9%). Most participants were between 25 and 44 years old (n = 157; 66.3%). The age distribution for the full sample ranged from 18 to 60 years, with 6.8% aged 18–24, 19.0% aged 45–54, and 8.0% aged 55–60.

To assess LMI, we included all participants who completed at least one wave of data collection. Data were collected at three time points (T1, T2, T3) across four instruments: the PSS-10, MBI-ES, and the Positive and Negative Affect subscales of the PANAS. The analytic sample consisted of 237 participants. Of these, 93 participants completed all three waves for the PSS-10, 90 for the MBI-ES, and 91 for both the Positive and Negative Affect subscales. In addition, 46 participants completed only T1 and T2, and 94 to 95 participants completed only T1 and T3, depending on the instrument. A small number of participants completed T1 only (ranging from four to seven per instrument), and very few exhibited other, less common missing data patterns. The consistency of these patterns across all four measures indicates moderate attrition over time, while retaining sufficient overlap across time points to support robust longitudinal Confirmatory Factor Analysis (CFA) models.

### Measures

#### Perceived Stress Scale

Using the Dutch PSS-10 (Cohen et al., 1983; Korten et al., 2017; van Eck et al., 1996), participants rated the frequency of ten feelings experienced during the past month. Example items include ‘feeling upset because of something that happened unexpectedly’ and ‘feeling unable to cope with all the things I have to do’. Response options ranged from 1 (never) to 5 (very often).

#### Maslach Burnout Inventory, Educators Survey

Emotional exhaustion was assessed with the Dutch MBI-ES (Soenens et al., 2012), which includes nine items referring to feelings experienced during the past two to three months. Example items include ‘feeling mentally exhausted by my work’ and ‘feeling empty at the end of the workday’. Responses were given on a Likert-type scale ranging from 1 (not at all) to 5 (very much).

#### Positive and Negative Affect Schedule

The Dutch PANAS (Watson et al., 1988) asked participants to indicate how much they had experienced various emotions during the past six weeks, using a scale from 1 (not at all) to 5 (very much). Each subscale consisted of ten items. Example Positive Affect items include ‘interested’, ‘excited’, and ‘proud’. Example Negative Affect items include ‘distressed’, ‘upset’, and ‘guilty’.

### Models and analyses

All analyses were conducted in Mplus Version 7 using full information maximum likelihood estimation (FIML). CFA was used to examine the factor structure and LMI of each scale. Model fit was evaluated using the chi-square test (χ^2^), Root Mean Square Error of Approximation (RMSEA), Standardized Root Mean Square Residual (SRMR), and Comparative Fit Index (CFI). Following common guidelines, adequate fit was indicated by χ^2^ p > 0.05, CFI > 0.90, and RMSEA and SRMR < 0.08 (Schreiber et al., 2006; Ullman & Bentler, 2012). These cut-offs are arbitrary and were treated as rules of thumb; therefore, all fit indices were considered jointly, and model evaluation was based on the overall pattern of results. To avoid model misspecification, we included design-driven correlated residuals between time-specific observations of the same items (Cole et al., 2007).

#### Factor structure

First, we examined whether the factor structures proposed in the literature for each of the three scales could be replicated in the present dataset. These analyses were conducted separately for each scale using the T1 data.

For the PSS-10, the developers proposed a unidimensional factor structure (Cohen & Williamson, 1988), a specification also adopted in studies using the Dutch version (Korten et al., 2017; Van Kerkhoven et al., 2022). Accordingly, this was the model tested in the present study.

The Emotional Exhaustion scale is part of the Maslach Burnout Inventory, Educators Survey (MBI-ES; Soenens et al., 2012). Both the original MBI manual (Maslach & Jackson, 1986) and the paper introducing the Dutch version (Soenens et al., 2012) treat the MBI subscales as separate, unidimensional constructs scored independently. Therefore, we evaluated a unidimensional model for the Emotional Exhaustion subscale.

The Positive and Negative Affect Schedule (PANAS) consists of two factors: Positive Affect and Negative Affect (Watson et al., 1988). Recent studies suggest that the optimal model includes two distinct factors. Within each of these factors, there are correlated error terms between certain sets of items (Engelen et al., 2006; Wedderhoff et al., 2021). This specification is based on Zevon and Tellegen’s (1982) finding that affect items cluster into content categories within the positive and negative affect dimensions (e.g., attentive, interested, and alert form the ‘attentive’ category; hostile, angry, and irritated form the ‘angry’ category). Therefore, we evaluated a one-factor model with correlated errors for each subscale.

#### LMI

To examine LMI across the three measurement occasions, we conducted a series of CFAs, each imposing additional constraints on model parameters.

In the first step, we tested configural invariance, which requires the same factor structure at all time points (i.e., the same items loading on the same factors). The factor loading of the first item was fixed to 1 by default (unit loading identification, ULI), and all other parameters were freely estimated across time.

In the second step, we tested metric invariance by constraining both the factor structure and factor loadings to be equal across time points.

Next, we tested scalar invariance by additionally constraining the item intercepts to be equal. The mean of the latent variable was fixed to zero at T1 for model identification.

Finally, we tested residual invariance by constraining the error variances of the items, in addition to the factor structure, factor loadings, and item intercepts, to be equal across time. This residual invariance model was the most restrictive specification in our analysis.

As a test of LMI, we evaluated changes in model fit at each step against the guidelines proposed by Chen (2007) for smaller samples (total N ≤ 300). A significant χ^2^ difference test served as an initial indication of possible non-invariance. In addition, a decrease in CFI greater than –0.005 combined with an increase in RMSEA greater than 0.010 was taken as further evidence of non-invariance. The cut-off for change in SRMR depends on the level of invariance being tested. For metric invariance, an increase in SRMR greater than 0.025 was considered problematic. For scalar and residual invariance, the criterion was stricter: an increase in SRMR greater than 0.005 was viewed as concerning.

These cut-offs were treated as rules of thumb and interpreted as part of an overall pattern rather than in isolation. We considered full LMI to be violated only when multiple indicators suggested non-invariance. In such cases, we tested whether partial invariance could be achieved by relaxing a subset of constraints (e.g., allowing one or more factor loadings to vary over time while keeping the remaining loadings constrained). In line with Byrne et al.’s (1989) recommendation, we required that at least two indicators per factor remain invariant.

In line with prior research highlighting the distinctiveness of the two affective dimensions, we analyze the Negative Affect and Positive Affect subscales of the PANAS separately (e.g., Crawford & Henry, 2004), resulting in four separate longitudinal CFA sequences, one for each of the following scales: PSS-10, MBI-ES, Negative Affect, and Positive Affect.

## Results

Table 2 reports the means, standard deviations, range, and omegas of each scale for all measurement occasions. The reliability for each of the scales was high and remained stable throughout the observation window. Appendix A has the standardized factor loadings (and their related standard errors) for the items in all scales.

**Table 2:** Means, Standard Deviations, Range, and Reliabilities for all Scales at T1, T2, and T3.


SCALE	T1			T2			T3		
		
	*M(SD)*	RANGE	ω	*M(SD)*	RANGE	ω	*M(SD)*	RANGE	ω

PSS ^a^	2.72(0.64)	1–5	0.90	2.65(0.59)	1–5	0.89	2.55(0.71)	1–5	0.94

EEX ^a^	2.71(0.84)	1–5	0.89	2.65(0.83)	1–5	0.90	2.58(0.88)	1–5	0.91

NA ^b^	2.55(0.62)	1–5	0.80	2.49(0.66)	1–5	0.85	2.38(0.68)	1–5	0.86

PA ^b^	3.40(0.57)	1–5	0.87	3.45(0.55)	1–5	0.87	3.49(0.62)	1–5	0.90


Note. PSS = Perceived Stress Scale; EEX = Maslach Burnout Inventory, Educators Survey, Emotional Exhaustion subscale; NA = PANAS, Negative Affect subscale; PA = PANAS, Positive Affect subscale; ω = McDonald’s omega. ^a^ N for PSS and EEX = 237. ^b^ N for NA and PA = 236.

### Factor structure

Table 3 presents the model fit of the unidimensional factor structures that were evaluated.

**Table 3:** Goodness-of-Fit Statistics for the Confirmatory Factor Analyses testing the Unidimensional Structure of all Scales, T1.


SCALE	*χ*^2^ (*df*)	*p*	RMSEA [90% CI]	SRMR	CFI

PSS ^a^	91.57 (35)	<0.001	0.083 [0.062, 0.103]	0.040	0.948

EEX ^a^	87.45 (27)	<0.001	0.097 [0.075, 0.120]	0.041	0.939

NA ^b^	62.02 (27)	<0.001	0.074 [0.050, 0.099]	0.054	0.949

PA ^b^	78.01 (26)	<0.001	0.092 [0.069, 0.116]	0.045	0.939


*Note*. PSS = Perceived Stress Scale; EEX = Maslach Emotional Exhaustion subscale; NA = PANAS Negative Affect subscale; PA = PANAS Positive Affect subscale; *df* = degrees of freedom; RMSEA = root mean square error of approximation; CI = confidence interval; SRMR = standardized root mean square residual; CFI = comparative fit index. ^a^ *N* for PSS and EEX = 237. ^b^ *N* for NA and PA = 236.

For all four scales, the unidimensional model fit reasonably. The estimates for SRMR and CFI were excellent in each CFA (SRMR < 0.08, CFI > 0.90), while RMSEA approached acceptability. For the PSS-10, Emotional Exhaustion, and Positive Affect, the estimate for RMSEA slightly surpassed the proposed cut-off of 0.08, but the lower bound of the confidence interval was satisfactory in all three cases. For Negative Affect, only the upper bound of the RMSEA confidence interval surpassed the proposed cut-off.

Overall, these results support the validity of the unidimensional structure of the PSS-10, the Emotional Exhaustion subscale of the MBI-ES, and the PANAS.

### LMI

The next step was to test the LMI of each scale by examining changes in model fit indices as progressively stricter constraints were imposed. Tables 4, 5, 6, 7 report all measurement invariance results. Given the factor structure results described above, we employed a unidimensional factor model for each latent variable.

**Table 4:** Model Fit Indices for the Longitudinal Configural, Metric, Scalar, Residual, and Partial Residual Invariant Models for Perceived Stress Scale (PSS-10).


LEVEL	GOODNESS-OF-FIT				MODEL COMPARISON				
	
	*χ*^2^ (*df*) ^*P*^	RMSEA [90% CI]	SRMR	CFI	REF. MODEL	Δ*χ*^2^ (Δ*df*) ^*P*^	ΔRMSEA	ΔSRMR	ΔCFI

Configural	598.36 (372) ***	0.051 [0.043, 0.058]	0.065	0.916					

Metric	615.90 (390) ***	0.049 [0.042, 0.057]	0.074	0.916	Configural	17.54 (18) ^n.s.^	–0.002	+0.009	+0.000

Scalar	634.83 (408) ***	0.048 [0.041, 0.056]	0.074	0.916	Metric	18.93 (18) ^n.s.^	–0.001	+0.000	+0.000

Residual	685.18 (428) ***	0.050 [0.043, 0.057]	0.093	0.905	Scalar	50.34 (20) ***	+0.002	+0.019	–0.011

Partial residual	655.82 (422) ***	0.048 [0.041, 0.055]	0.083	0.913	Scalar	20.99 (14) ^n.s.^	+0.000	+0.009	–0.003


*Note*. *df* = degrees of freedom; RMSEA = root mean square error of approximation; CI = confidence interval; SRMR = standardized root mean square residual; CFI = comparative fit index; Ref. model = reference model; *N* = 237. *** *p* < 0.001; *^n^*^.s.^ = not significant.

#### Perceived Stress Scale

Table 4 shows the results of the measurement invariance tests for the PSS-10. The fit of the configural model was excellent (RMSEA and SRMR < 0.08, CFI > 0.90), as was the fit of the metric invariant model. The metric invariant model fit the data only slightly worse than the configural model, with a non-significant change in χ^2^, a small decrease in RMSEA, a slight increase in SRMR, and no change in CFI. This supports the metric measurement invariance of the PSS-10. The scalar invariant model fit similarly, and no significant deterioration in fit was detected when compared to the metric invariant model, supporting scalar invariance over time. Finally, the residual invariant model did reflect fit deterioration, with a significant χ^2^ difference test, and changes in SRMR and CFI that are greater than the proposed cut-offs (ΔSRMR > +0.005 and ΔCFI < –0.005). Taken together, three out of four model fit indices indicated residual non-invariance. Therefore, we tested if we could reach partial residual invariance by progressively relaxing the constraints for one residual variance at a time while evaluating the change in model fit with every step. We started with the item that demonstrated the largest differences in residual variances over time and worked our way down until the change in model fit was acceptable. Eventually, we reached partial residual invariance after releasing the constraints for three items (PSS2, PSS7, PSS9) (Δχ^2^ *p* > 0.05, ΔRMSEA < 0.010, ΔSRMR < 0.005, ΔCFI < –0.005, compared to the scalar invariant model).

Altogether, these results indicate support for the full configural, metric, and scalar LMI, and partial residual LMI of the Dutch version of the PSS-10.

#### Emotional exhaustion

For Emotional Exhaustion, Table 5 shows the model fit indices. The fit of the configural model was particularly good (RMSEA and SRMR < 0.08, CFI > 0.90). Constraining the factor loadings to be equal over time led to a similar fit, and no significant deteriorations were observed. This supports the metric invariance of the Emotional Exhaustion subscale. The additional constraints for the intercepts also did not cause unacceptable deteriorations in model fit, supporting scalar invariance. The full residual invariant model maintained an adequate fit, with only the change in SRMR slightly higher than the proposed cut-off of 0.005. Since the other fit indices did not display signs of non-invariance, we conclude that these results support the full configural, metric, scalar, and residual LMI of the Emotional Exhaustion subscale.

**Table 5:** Model Fit Indices for the Longitudinal Configural, Metric, Scalar, and Residual Invariant Models for Maslach Burnout Inventory, Educators Survey (MBI-ES), Emotional Exhaustion subscale.


LEVEL	GOODNESS-OF-FIT				MODEL COMPARISON				
	
	*χ*^2^(*df*) ^*P*^	RMSEA [90% CI]	SRMR	CFI	REF. MODEL	Δ*χ*^2^ (Δ*df*) ^*P*^	ΔRMSEA	ΔSRMR	ΔCFI

Configural	483.15 (294) ***	0.052 [0.044, 0.060]	0.069	0.923					

Metric	496.43 (310) ***	0.050 [0.042, 0.058]	0.074	0.925	Configural	13.28 (16) ^n.s.^	–0.002	+0.005	+0.002

Scalar	521.32 (326) ***	0.050 [0.042, 0.058]	0.076	0.921	Metric	24.89 (16) ^n.s.^	+0.000	+0.002	–0.004

Residual	540.63 (344) ***	0.049 [0.041, 0.057]	0.082	0.920	Scalar	19.30 (18) ^n.s.^	–0.001	+0.006	–0.001


*Note*. *df* = degrees of freedom; RMSEA = root mean square error of approximation; CI = confidence interval; SRMR = standardized root mean square residual; CFI = comparative fit index; Ref. model = reference model; *N* = 237. *** *p* < 0.001; ^n.s.^ = not significant.

#### Negative affect

Table 6 presents the results for Negative Affect. The fit of the configural invariant model approached adequacy, with CFI and SRMR close to the proposed cut-offs. Model fit remained stable for the metric invariant model, with no signs of non-invariance. However, constraining all item intercepts to be equal was too stringent and resulted in a significant *χ*^2^ difference test and a decrease in CFI beyond the proposed limit of –0.005. This combination of results led us to conclude that full scalar invariance was not present in this dataset and to investigate whether we could reach partial scalar invariance. Releasing the constraints for the intercepts of two items was necessary to reach partial scalar invariance (‘scared’ and ‘distressed’, which showed intercept changes from 2.51 to 2.14 and from 2.60 to 2.28, respectively). Finally, the fit of the residual invariant model was acceptable, without deteriorations from the partial scalar invariant model. This supports the residual LMI of the Negative Affect subscale.

**Table 6:** Model Fit Indices for the Longitudinal Configural, Metric, Scalar, and Residual Invariant Models Positive and Negative Affect Schedule (PANAS), Negative Affect subscale.


LEVEL	GOODNESS-OF-FIT				MODEL COMPARISON				
	
	*χ*^2^(*df*) ^*P*^	RMSEA [90% CI]	SRMR	CFI	REF. MODEL	Δ*χ*^2^ (Δ*df*) ^*P*^	ΔRMSEA	ΔSRMR	ΔCFI

Configural	557.11 (348) ***	0.050 [0.043, 0.058]	0.085	0.895					

Metric	579.46 (366) ***	0.050 [0.042, 0.057]	0.092	0.893	Configural	22.35 (18)^n.s.^	+0.000	+0.007	–0.002

Scalar	612.71 (384) ***	0.050 [0.042, 0.057]	0.093	0.885	Metric	33.25 (18) *	+0.000	+0.001	–0.008

Partial scalar	602.66 (380) ***	0.050 [0.042, 0.057]	0.093	0.888	Metric	23.21 (14) ^n.s.^	+0.000	+0.001	–0.005

Residual	621.62 (400) ***	0.048 [0.041, 0.056]	0.097	0.889	Partial scalar	18.96 (20) ^n.s.^	–0.002	+0.004	+0.001


*Note*. *df* = degrees of freedom; RMSEA = root mean square error of approximation; CI = confidence interval; SRMR = standardized root mean square residual; CFI = comparative fit index; Ref model = reference model; *N* = 236. *** *p* < 0.001; * p < 0.05; *^n^*^.s.^ = not significant.

#### Positive affect

We performed the same sequence of analyses for the Positive Affect subscale. The results are presented in Table 7. The configural model fit the data borderline adequately, with the CFI slightly below the proposed 0.90, and the SRMR slightly above 0.08. The model fit did not change significantly for the metric invariant model, nor the scalar or residual invariant models. The change in model fit indices remained within the proposed boundaries. Therefore, we conclude that this dataset supports the full LMI of the PANAS Positive Affect subscale.

**Table 7:** Model Fit Indices for the Longitudinal Configural, Metric, Scalar, and Residual Invariant Models Positive and Negative Affect Schedule (PANAS), Positive Affect subscale.


LEVEL	GOODNESS-OF-FIT				MODEL COMPARISON				
	
	*χ*^2^(*df*) ^*P*^	RMSEA [90% CI]	SRMR	CFI	REF. MODEL	Δ*χ*^2^ (Δ*df*) ^*P*^	ΔRMSEA	ΔSRMR	ΔCFI

Configural	601.62 (345) ***	0.056 [0.049, 0.064]	0.082	0.887					

Metric	619.28 (363) ***	0.055 [0.047, 0.062]	0.090	0.887	Configural	17.67 (18) ^n.s.^	–0.001	+0.008	+0.000

Scalar	635.63 (381) ***	0.053 [0.046, 0.060]	0.091	0.888	Metric	16.35 (18) ^n.s.^	–0.002	+0.001	+0.001

Residual	651.02 (401) ***	0.051 [0.044, 0.058]	0.093	0.890	Scalar	15.39 (20) ^n.s.^	–0.002	+0.002	+0.002


*Note. df* = degrees of freedom; RMSEA = root mean square error of approximation; CI = confidence interval; SRMR = standardized root mean square residual; CFI = comparative fit index; Ref model = reference model; *N* = 236. *** *p* < 0.001; *^n^*^.s.^ = not significant.

Overall, these results support the full configural, metric, partial scalar and residual LMI of this subscale.

## Discussion

In this study, we investigated the Dutch versions of three widely used well-being scales: the Perceived Stress Scale (PSS-10), the Emotional Exhaustion subscale of the Maslach Burnout Inventory (MBI-ES), and the Positive and Negative Affect Schedule (PANAS). Our aim was to examine the LMI of these scales using data from three consecutive assessment points collected over a six-month period.

### Factor structure

We began by testing whether the factor structure of each scale matched the structure proposed in the literature. The proposed unidimensional factor structure showed acceptable fit to the data for all three scales. These results are consistent with the developers’ guidelines (Cohen & Williamson, 1988; Maslach & Jackson, 1986; Watson et al., 1988) and with previous studies using the Dutch versions (Ceulemans et al., 2021; Korten et al., 2017; Laporte et al., 2022; Van den Berghe et al., 2013; Vermote et al., 2023). For the PANAS, we included correlated error terms following Zevon and Tellegen (1982), who proposed that items within each affect dimension cluster into content categories. This addition has been supported in prior research (Crawford & Henry, 2004) and, in our data, a model including correlated error terms also demonstrated good fit.

### LMI

Once the factor structures were confirmed, we tested four levels of LMI: configural, metric, scalar, and residual invariance.

For the Dutch PSS-10, we found no evidence against full LMI at the configural, metric, or scalar levels, in line with earlier findings for the English, German, and Turkish versions (Barbosa-Leiker et al., 2013; Isik Akin et al., 2024; Reis et al., 2019; Shields et al., 2016). The Chinese version was the first to test residual invariance and found support for it (Jiang et al., 2023). In our data, we observed partial residual invariance: the error variances for three items varied over time (‘Feeling unable to control the important things in my life’, ‘Being able to control irritations in my life’, and ‘Feeling angry because things were out of my control’).

For the Dutch Emotional Exhaustion subscale of the MBI-ES, our data supported full LMI. Previous studies on other language versions have generally reported full configural, metric, and residual invariance (e.g., Finnish, Mäkikangas et al., 2011; German, Turhan et al., 2021), although evidence for scalar invariance has been mixed. While the German version demonstrated full scalar invariance (Turhan et al., 2021), the Italian version showed scalar non-invariance (Giusti et al., 2022). In our data, we found no evidence against full scalar invariance.

For the Positive Affect subscale of the PANAS, our results indicated full LMI. Previous studies on this subscale have reported similar findings for configural and metric invariance in the English, Danish, and Chinese versions (Jønsson & Kähler, 2022; Schnitker et al., 2020; Xiang et al., 2023). Scalar invariance has been supported for the English and Danish versions (Jønsson & Kähler, 2022; Schnitker et al., 2020) but not for the Chinese version (Xiang et al., 2023). Residual invariance of the PANAS had not been examined before; our results showed no signs of non-invariance at any level. This study is also the first to investigate the LMI of the Negative Affect subscale. We found evidence for full configural and metric invariance, and partial scalar and residual invariance. Two items showed changes in intercepts over time: the intercepts for ‘scared’ and ‘distressed’ declined over the six-month period.

## Contributions and implications

This study makes several contributions to the literature.First, LMI of the PSS-10, the Emotional Exhaustion subscale of the MBI, and the PANAS has received limited attention, and very few prior studies have examined employee samples. Those that have done so show notable limitations. For example, Giusti et al. (2022) used only two waves of data collection with a long interval between them (16 months) and did not test for residual invariance. Mäkikangas et al. (2011) also used two waves and claimed residual invariance without testing for scalar invariance. Jønsson and Kähler (2022), while employing three waves, did not test for residual invariance and relied on a shortened version of the scale.The present study addresses these gaps by examining all four levels of LMI in an employee sample, using three waves of data collection spaced two months apart.

Our findings extend the evidence base for the psychometric properties of these scales and support their value in well-being research and practice. Demonstrating at least partial LMI for each scale means that temporal changes in latent means and in structural relationships with other constructs can be interpreted more confidently as reflecting real changes in the underlying constructs. This strengthens the ability of researchers and practitioners to track developmental trends in well-being and to monitor the effects of well-being interventions using these instruments.

## Limitations and future directions

Despite these contributions, several limitations should be noted.

First, our sample size was relatively small, which limited statistical power. We accounted for this by applying Chen’s cut-off criteria for small samples (N ≤ 300) (Chen, 2007). Future research should examine whether our findings replicate in larger samples.

Second, for the PSS-10 we adhered to the factor structure proposed by the original developers (Cohen et al., 1983) and used in other studies with the Dutch version (Ceulemans et al., 2021; Dijkstra et al., 2017; Van Kerkhoven et al., 2022). However, recent research has explored alternative structures, including bifactor and hierarchical models, for the German and English versions (Reis et al., 2019; Yılmaz Koğar & Koğar, 2024). Future studies could investigate whether such alternative models also apply to the Dutch PSS-10.

A further limitation concerns the spacing of the measurement occasions. The two-month intervals used in this study may influence the likelihood of detecting LMI. Shorter intervals might reduce the chance of substantive change in how participants interpret items, thereby increasing the probability of finding invariance, whereas longer intervals could allow for more substantial changes in item interpretation and reduce invariance. Consequently, the present findings should be interpreted considering the specific time frame used. Future research could explore whether invariance patterns for these scales differ when shorter or longer intervals are applied.

## Conclusion

In this sample, our results indicate at least partial LMI of the Dutch Perceived Stress Scale, Emotional Exhaustion subscale of the Maslach Burnout Inventory, and Positive and Negative Affect Schedule over three consecutive time points. This suggests that these scales measure their respective constructs similarly over six months. We can use these scales to investigate structural relationships with other constructs, latent mean differences, item uniqueness, and measurement errors over different assessment points. Additionally, we can more confidently attribute changes in their scores to actual changes in the construct over time.

## Additional File

The additional file for this article can be found as follows:

10.5334/pb.1528.s1 Supplementary File. Appendices A to D.

## Data Availability

This study is part of a larger project registered in the ISRCTN registry (ISRCTN61170784).
