# Supplementary material for: Testing Longitudinal Measurement Invariance of the Dutch PSS-10, MBI-ES, and PANAS in a Bimonthly 6-Month Panel
Source: Psychol Belg. 2026 May 25;66(1):65–80. doi: 10.5334/pb.1528 (PMC13220742; doi:10.5334/pb.1528)
Supplement: Supplementary File. — Appendices A to D. [file pb-66-1-1528-s1.pdf]

Appendix A

Standardized factor loadings and standard errors

		Est.	SE
PSS	item 1	0.747	0.028
	item 2	0.792	0.025
	item 3	0.712	0.031
	item 4	-0.543	0.040
	item 5	-0.674	0.035
	item 6	0.717	0.031
	item 7	-0.653	0.035
	item 8	-0.726	0.030
	item 9	0.603	0.037
	item 10	0.758	0.029
EEX	item 1	0.789	0.026
	item 2	0.771	0.027
	item 3	0.603	0.037
	item 4	0.678	0.034
	item 5	0.830	0.025
	item 6	0.667	0.035
	item 7	0.580	0.038
	item 8	0.589	0.039
	item 9	0.739	0.030
NA	item 1	0.502	0.045
	item 2	0.499	0.049
	item 3	0.615	0.046
	item 4	0.524	0.048
	item 5	0.572	0.046
	item 6	0.456	0.049
	item 7	0.433	0.054
	item 8	0.502	0.045
	item 9	0.573	0.045
	item 10	0.615	0.045
PA	item 1	0.655	0.041
	item 2	0.620	0.037
	item 3	0.530	0.042
	item 4	0.793	0.029
	item 5	0.740	0.031
	item 6	0.651	0.036
	item 7	0.621	0.040
	item 8	0.442	0.047
	item 9	0.693	0.037
	item 10	0.589	0.039

Note. PSS = Perceived Stress Scale; EEX = Maslach Emotional Exhaustion subscale; NA = PANAS Negative Affect subscale; PA = PANAS Positive Affect subscale; the estimates are standardized (STDYX) loadings and their standard errors, based on the metric invariance model.

Appendix B

Perceived Stress Scale (PSS)

Onderstaande vragen gaan over uw gevoelens en gedachten in de afgelopen maand.

Bij elke vraag willen we graag weten hoe vaak u zich op een bepaalde manier voelde.

	Nooit	Bijna nooit	Soms	Vaak	Zeer vaak
In de afgelopen maand, hoe vaak was u overstuur vanwege iets dat onverwacht gebeurde?	( )	( )	( )	( )	( )
In de afgelopen maand, hoe vaak voelde u dat u niet in staat was controle te hebben over de belangrijke dingen in uw leven?	( )	( )	( )	( )	( )
In de afgelopen maand, hoe vaak voelde u zich nerveus en “gestrest”?	( )	( )	( )	( )	( )
In de afgelopen maand, hoe vaak voelde u zich zelfverzekerd over uw vermogen om met persoonlijke problemen om te gaan?	( )	( )	( )	( )	( )
In de afgelopen maand, hoe vaak voelde u dat dingen gingen zoals u wilde?	( )	( )	( )	( )	( )
In de afgelopen maand, hoe vaak voelde u dat u niet kon omgaan met (of het hoofd kon bieden aan) alle dingen die u moest doen?	( )	( )	( )	( )	( )
In de afgelopen maand, hoe vaak kon u uw irritaties in uw leven onder controle houden?	( )	( )	( )	( )	( )
In de afgelopen maand, hoe vaak voelde u dat u greep had op de dingen?	( )	( )	( )	( )	( )
In de afgelopen maand, hoe vaak was u boos omdat dingen buiten uw controle waren?	( )	( )	( )	( )	( )
In de afgelopen maand, hoe vaak voelde u dat moeilijkheden zich zo hoog opeen stapelden dat u ze niet kon overwinnen?	( )	( )	( )	( )	( )

Appendix C

Maslach Burnout Inventory, Educators Survey – Emotional exhaustion subscale (EEX)

Als terugkijk naar **de laatste 2 à 3 maanden**, dan …

	Helemaal niet zo				Helemaal wel zo
voelde ik mij mentaal uitgeput door mijn werk.	( )	( )	( )	( )	( )
voelde ik mij leeg op het eind van een werkdag.	( )	( )	( )	( )	( )
voelde ik mij vermoeid als ik 's morgens opsta en aan een nieuwe werkdag moet beginnen.	( )	( )	( )	( )	( )
voelde ik een ganse dag werken met leerlingen aan als een zware belasting.	( )	( )	( )	( )	( )
voelde ik mij opgebrand door mijn werk.	( )	( )	( )	( )	( )
voelde ik mij gefrustreerd in mijn werk.	( )	( )	( )	( )	( )
had ik het gevoel dat ik me te veel inzet voor school.	( )	( )	( )	( )	( )
bezorgde werken met mijn leerlingen mij te veel stress.	( )	( )	( )	( )	( )
voelde ik mij alsof ik aan het eind van mijn krachten ben.	( )	( )	( )	( )	( )

Appendix D

Positive and Negative Affect Schedule (PANAS)

Gelieve voor elk van de volgende emoties aan te geven in welke mate je deze emotie gedurende de afgelopen 6 weken hebt ervaren. Gelieve een getal tussen 1 (helemaal niet ervaren) en 5 (heel sterk ervaren) te omcirkelen.

In de afgelopen 6 weken voelde ik me…

	Helemaal niet ervaren	Een beetje	Niet meer of minder dan anders	Sterk	Heel sterk ervaren
Enthousiast	( )	( )	( )	( )	( )
Angstig	( )	( )	( )	( )	( )
Geïnteresseerd	( )	( )	( )	( )	( )
Kwaad	( )	( )	( )	( )	( )
Vastberaden	( )	( )	( )	( )	( )
Gestresseerd	( )	( )	( )	( )	( )
Geïrriteerd	( )	( )	( )	( )	( )
Zenuwachtig	( )	( )	( )	( )	( )
Opgewekt	( )	( )	( )	( )	( )
Actief	( )	( )	( )	( )	( )
Sterk	( )	( )	( )	( )	( )
Vijandig	( )	( )	( )	( )	( )
Beschaamd	( )	( )	( )	( )	( )
Fier	( )	( )	( )	( )	( )
Schuldig	( )	( )	( )	( )	( )
Attent	( )	( )	( )	( )	( )
Geïnspireerd	( )	( )	( )	( )	( )
Nerveus	( )	( )	( )	( )	( )
Bedroefd	( )	( )	( )	( )	( )
Alert	( )	( )	( )	( )	( )
Depressief	( )	( )	( )	( )	( )
